# Reasons to not correct for leaching in TBI; Reply to Lind et al. (2022)

**DOI:** 10.1002/ece3.10133

**Published:** 2023-06-13

**Authors:** Judith M. Sarneel, Janna M. Barel, Sarah Duddigan, Joost A. Keuskamp, Ada Pastor, Taru Sandén, Gesche Blume‐Werry

**Affiliations:** ^1^ Department of Ecology and Environmental Science Umeå University Umeå Sweden; ^2^ Aquatic Ecology & Environmental Biology, Faculty of Science, Radboud Institute for Biological and Environmental Sciences Radboud University Nijmegen Nijmegen The Netherlands; ^3^ Soil Research Centre and Department of Geography & Environmental Science University of Reading Reading UK; ^4^ Ecology & Biodiversity Group, Institute of Environmental Biology Utrecht University Utrecht The Netherlands; ^5^ Biont Research Utrecht The Netherlands; ^6^ GRECO, Institute of Aquatic Ecology University of Girona Girona Spain; ^7^ Department for Soil Health and Plant Nutrition Austrian Agency for Health and Food Safety (AGES) Vienna Austria

## Abstract

We believe that correcting for leaching in (terrestrial) litterbags studies such as the Tea Bag Index will result in more uncertainties than it resolves. This is mainly because leaching occurs in pulses upon changes in the environment and because leached material can still be mineralized after leaching. Furthermore, amount of material that potentially leaches from tea is comparable to other litter types. When correcting for leaching, it is key to be specific about the employed method, just like being specific about the study specific definition of decomposition.

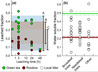

## 1 INTRODUCTION

**FIGURE 1:**
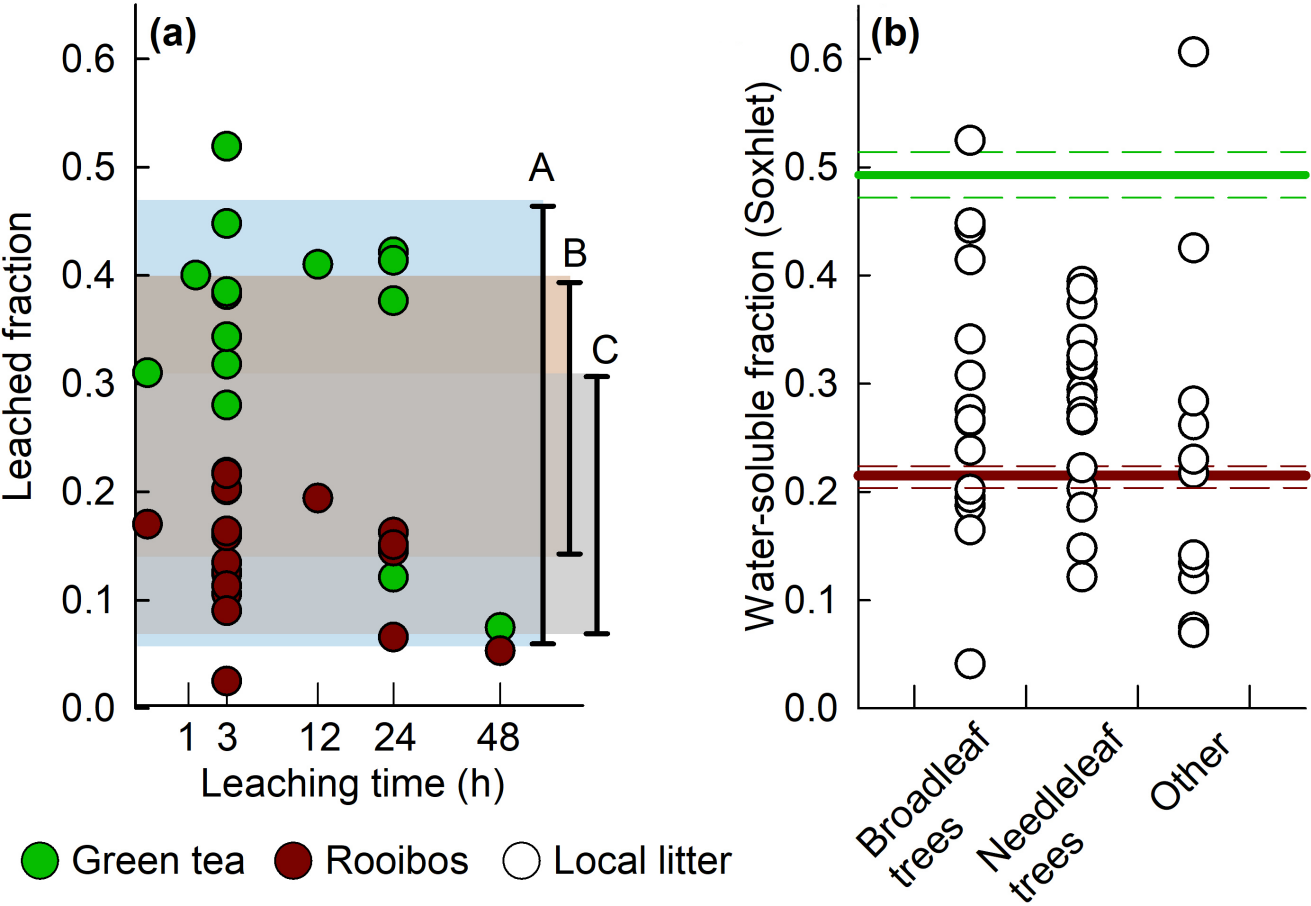
(a) variation in leaching estimates of rooibos and green tea in literature sorted from short to long incubation durations on a square root transformed scale to enhance visibility. Studies included Djukic et al. (2018) leaching 3 min at 100°C, Pouyat et al. (2017) leaching 80 min at 60°C (only green tea), Seelen et al. (2019), leaching 3 h under outdoor conditions (9.5–14°C), Lind et al. (2022) leaching 3 h outdoor measurements, and at 8, 19 and 60°C in the laboratory, Blume‐Werry et al. (2021), leaching 12 h at 25°C, Mori et al. (2021) leaching 24 h at 3, 15 and 25°C and Thomas et al., (2023) leaching 24 and 48 h under outdoor conditions and 24 h at room temperature (±20°C). Shaded areas represent the ranges of leaching of local litter reported in (A) Jiang et al. (2016), (B) Friesen et al. (2018), and (C) Xiong and Nilsson (1997). (b) Variation in water soluble fraction in tea and other plant material (leachable in a Soxhlet extraction; Harmon, 2016) with the red and the green line representing the initial water‐soluble fraction of rooibos and green tea respectively and their standard deviation (Keuskamp et al., 2013). The category ‘other’ includes graminoids, some lichens but no forbs.

During litter decomposition, a fraction of the water‐soluble components of the litter is quickly dissolved (leached) into the water that is available in the environment. Besides leaching, litter decomposition is driven by fragmentation, (UV)‐bleaching and microbial activity. Many studies quantify litter decomposition by measuring mass‐loss rates of incubated plant material, which inherently integrate the biotic and abiotic processes that drive litter decomposition. Although some studies argued that leaching is an artifact of mass‐loss studies due to pre‐drying of the material, this is likely mostly a problem in studies that use dried aquatic plants (Boulton & Boon, 1991). In 2013, the Tea Bag Index (TBI) was published, which is an easy method that uses tea bags as standardized alternative to litter bags filled with local litter (Keuskamp et al., 2013). Recently, Lind et al. (2022) and others (Figure 1a) used TBI to explicitly address and quantify leaching. In addition, frameworks like the Microbial Efficiency‐Matrix Stabilization (Cotrufo et al., 2013) and increased interest in fluxes of dissolved organic matter from soils (Cleveland et al., 2004; Shumilova et al., 2019) further highlight the role of leaching during litter decomposition. Mechanistic studies such as presented by Lind et al. (2022) contribute to an increased understanding of the factors that drive leaching losses during litter decomposition. While Lind et al. (2022) call for a calculated leaching correction when using the Tea Bag Index, we believe that this would result in more uncertainties than it resolves, especially in terrestrial TBI and other mass‐loss based studies. As we will explain below, this is mainly because (primary) leaching occurs in pulses upon changes in the environment and because leached material can still be mineralized after leaching. Furthermore, the amount of material that potentially leaches from tea is comparable to other litter types. Finally, it introduces a high degree of methodological heterogeneity (such as in duration of leaching tests, in other environmental conditions or in applied calculations). As a result, correcting for leaching hampers the interpretation, decreases comparability across studies and increases the complexity of the TBI that is designed to be a standardized and simple method.

## 2 DEFINITIONS

The definition of decomposition may vary due to the aim of the study (Benfield et al., 2017) and the methods used. However, “litter decomposition” is often (explicitly or implicitly) used as an umbrella concept for processes leading to mass loss of litter, including fragmentation, leaching of primary material, bleaching and biochemical degradation (Campbell et al., 2009; Smith & Smith, 2003). Leaching refers to the loss of soluble compounds by dissolving primary material or secondary material created during breakdown. “Mineralization” is frequently used as an alternative for a more narrow definition of decomposition that places the biological activity in the center. Studies that measure mass loss of litter, implicitly or explicitly integrate more processes than mineralization. Since the TBI is a method based on mass‐loss from tea as a plant litter, we use the wider definition of ‘litter decomposition’, including fragmentation, leaching, bleaching and mineralization. However, due to the small mesh of buried tea bags, fragmentation and bleaching may not contribute much to the mass losses observed in TBI as compared to studies using litter bags with a wider mesh size placed on top of the soil. Like Lind et al. (2022), Benfield et al. (2017) and Boulton and Boon (1991), we argue to be explicit about definitions in order to minimize confusion in the scientific discussion.

## 3 LEACHING IN TEA

There are two common approaches to account for leaching in mass loss studies, which both may have their own implications. The first is a posteriori mathematical correction of the initial mass based on a (local) measurement of the mass loss during a short period of time (as in Lind et al., 2022; Seelen et al., 2019). Alternatively, litterbags are soaked a priori, before incubation to remove most of the water‐soluble material (Elwood et al., 1981). In this comment, we argue against a generalized application of either type of leaching corrections in TBI.

The TBI consists of burying two types of tea bags as an easy, standardized alternative for litter bags filled with local litter (Keuskamp et al., 2013). The mass loss after ca. 3 months is used to parameterize the litter decomposition curve and obtain a litter decomposition rate that estimates the mass loss of the soluble and hydrolysable compounds in rooibos tea. Although we do not claim that the tea used in TBI is completely natural, it resembles the chemical and structural complexity of local litter better than other standard materials (e.g., cotton strips or wooden sticks), Moreover, the water‐soluble fraction of tea used in TBI (the total of leachable material in a soxhlet extraction) is well in range with other litter (Figure 1b; Harmon, 2016). We therefore disagree with the statement of Lind et al. (2022) that ‘initial leaching of water‐soluble compounds may therefore be even higher in the tea bag decomposition substrates than for intact leaves of traditional litterbag studies’. On average, leaching in rooibos and green tea is within the ranges reported in the three review studies to our knowledge available on leaching (mass loss of 14%–40%, 5.7%–47.2% and 7%–31%, respectively; Friesen et al., 2018; Jiang et al., 2016; Xiong & Nilsson, 1997; Figure 1). From the leaching measurements done on tea so far, it also becomes clear that the duration, temperature and moisture availability of the soil cause considerable variation in the amount that is leached. This means that factors that determine leaching overlap with, and are hard to separate from those that determine litter quality and mineralization (Shumilova et al., 2019).

## 4 REASONS AGAINST AN A POSTERIORI LEACHING CORRECTION

**FIGURE 2:**
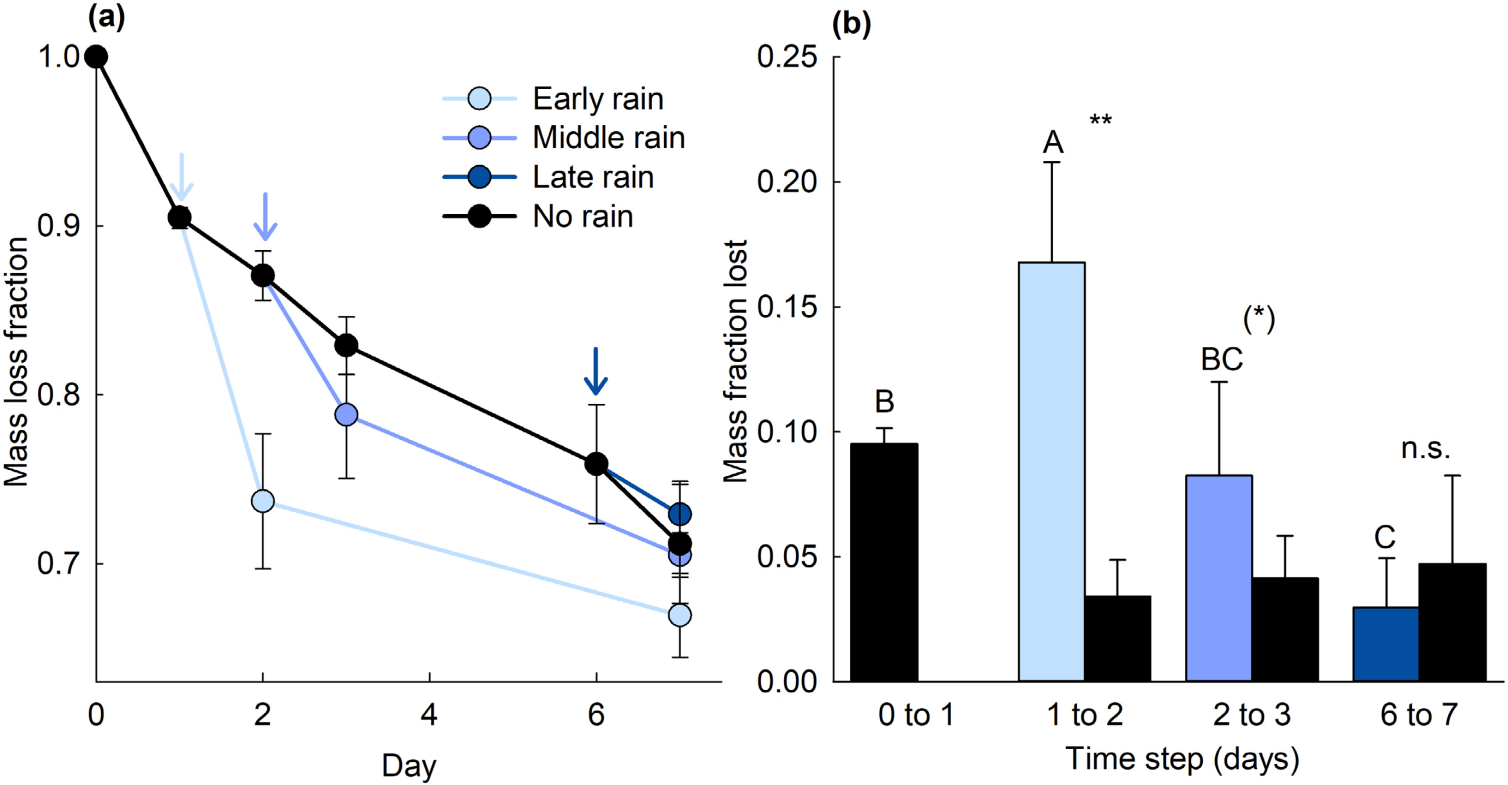
(a) Measured mass loss of green tea after identical rainfall events in different rain‐timing treatments (*n* = 4) on wet soil (field capacity). Arrows (with corresponding colors) indicate when 60 ml water was added to the pots. Lipton green tea bags (biodegradable bags) were individually placed at 4 cm depth in pots (7.5 × 7.5 × 7 cm) containing a 15:2 mixture of potting soil (k‐jord; NPK 14–7‐15, Hasselfors Garden AB) and quartz sand (Tärnsjö Grus AB). Soil at field capacity had a soil moisture of 53.2%, whereas flooded soils had a soil moisture of 63.0%. After incubation in a dark climate room at 18°C, the bags were cleaned without water, dried (at least 48 h at 65°C) and weighed to assess mass loss. (b) Mass lost in different time steps calculated as the differences between the mass observed before and after the rain additions (or placement in the soil for the initial mass loss). Letters indicate significant differences in leached amounts (capitals) tested with a one‐way ANOVA and Tukey post‐hoc tests. An asterisks (**p < .001, .001 < *p* < .05, (*), .05 > *p* > .1) indicate whether the mass loss in the rain treatment significantly differed from the mass loss observed in the control treatment without rain which was tested with *t*‐test in R version 4.2.2 (R Core Team, 2022).

Lind et al. (2022) advocate that correcting litter decomposition rates for leaching would improve the TBI method (and implicitly other litterbag studies). The TBI method intends to obtain a standardized, easy measurement of mass losses and introducing a leaching correction would complicate its practical use as well as introduce uncertainties in its’ interpretation. Firstly, leaching is a continuous process and both un‐digested starting products (primary leaching) and products resulting from degradation (secondary leaching) can leach when conditions allow (Figure 2; see the Appendix 1). Franklin et al. (2020) found that a heavy rainfall leached only 3%–5% of the potential leachable material (determined by soaking 24 for hours). We found that a rain event only 24 h after the start of incubation leached significantly more (potentially primary material) from green tea than was observed as leaching in soils watered to field capacity (treatment effect in one‐way ANOVA; *F*_3,12_ = 15.06, *p* < .001 and post‐hoc *p* = .020; Figure 2). This makes it likely that additional primary material can leach if conditions (temperature, moisture) change shortly after the start of incubation (Figure 2). Shorter incubation durations likely increase the probability of this happening. Lind et al. (2022) propose to correct for primary leaching using a three‐hour interval incubation. We argue that primary leaching may instead occur in stochastic pulses (e.g. a rain event, temperature increases; Figure 2). Consequently, the duration of the period in which primary leaching takes place may be hard to estimate and may differ between ecosystems, between seasons within the same ecosystem, or between days or weeks due to variation in temperature and water availability (as shown in Lind et al., 2022) and other unpredictable precipitation events. This may interfere with timescales in which mineralization causes measurable mass loss. In aquatic systems, leaching as an initial event is possible to quantify (Elwood et al., 1981; Gessner et al., 1999; Seelen et al., 2019). However, in these systems, the duration of leaching measurements is also unstandardised (although frequently 24 h) and correcting mass losses for leaching is relatively uncommon (Benfield et al., 2017; Robbins et al., 2022).

A second uncertainty introduced by the proposed leaching correction is that the leached material is not necessarily exempt from further microbial decomposition. In fact, a large part of the leached components will be elsewhere mineralized after leaching (Cleveland et al., 2004; Deng et al., 2017).

Thirdly, Lind et al. (2022) convincingly show that leaching depends on specific conditions of the environment. This questions the use of leaching measurements from one location or time point (because temperature and moisture changes over time) to correct mass loss at another location (as in Lind et al., 2022; Seelen et al., 2019). If there is a conceptual and practical need for a leaching correction, this should be done under exactly the same settings as the incubation (Lind et al., 2022; Wang et al., 2021). This in turn, requires educated guesses on the duration of the leaching period (Figure 2), the timescales at which mineralization causes mass losses as well as the relative importance of maximum versus average moisture or temperature conditons for causing primary leaching. We believe that this will lead to a high degree of heterogeneity among measurements which hamper standardization and comparability.

Lastly, we question the underlying theoretical implications of an a posteriori leaching correction because such correction assumes that the leached material is no longer part of the litter. Especially when the suggestion of Lind et al. (2022) is followed and leaching is measured very locally, this introduces variation of starting material within one experiment (e.g. when comparing dryer and wetter locations). Moreover, such variation in initial litter composition (for instance in C:N ratios) will be hard to quantify (Schreeg et al., 2013). Therefore, an a posteriori correction requires an implicit assumption that such differences are not important for the final mass loss. Yet, decomposition depends on litter quality (Djukic et al., 2018; Fortino et al., 2020) and hence, from a theoretical point of view, such differences may not be trivial. When mass loss processes do not exclude leaching a posteriori, starting material is equal across locations and instead, differences in initial leaching (when measured) can be used to understand the mass loss dynamics.

## 5 A PRIORI SOAKING TREATMENTS

Comparable to other litter bag studies (Elwood et al., 1981; Fortino et al., 2020; Grimmett et al., 2012; Halvorson et al., 2019), a number of TBI studies address leaching by soaking the tea bags with water before incubating them for TBI (Blume‐Werry et al., 2021; Kotze & Setälä, 2022; Pouyat et al., 2017; Toth et al., 2017, 2018). Pre‐leaching treatments range from ca 1.3 h (Pouyat et al., 2017) to 5 days (Fortino et al., 2020). Pre‐leaching may often extract more material from the tea than the environmental settings would. For instance, Lind et al. (2022) and Thomas et al. (2023) measured 7%–12% of green tea mass to be leached in outdoor soils, whereas >35% of the initial tea mass was leached in indoor treatments that resembled pre‐leaching treatments (Figure 1). Therefore, a soaking pre‐treatment could make the tea less comparable to local litter (Boulton & Boon, 1991). This in turn, may modify microbial activity that depends on litter quality. Blume‐Werry et al. (2021) explicitly tested the effect of pre‐leaching. They found that although the absolute magnitude of the mass losses changed, relative treatment effects did not. Hence, they conclude that this additional step in the protocol does not result in different study outcomes and hence is not needed. Moreover, calculating the TBI proxies would need the correction proposed by Seelen et al. (2019) using the pre‐leached fraction. Yet, TBI proxies of pre‐leached tea should not be compared to un‐pre‐leached tea which interferes with standardization of the method.

## 6 CONCLUSION

Lind et al. (2022) convincingly showed that the same factors (temperature and moisture) that affect mineralization can also drive differences in leaching, and flag for higher appreciation of this process in the TBI, mass‐loss and litterbag studies. Yet, making a mathematical correction of leaching part of the standardized TBI method is not feasible or desirable. It introduces more uncertainties than it solves and undermines the purpose of the method: standardization between studies. Instead, alternative methods like those that use variation in the solubility of different elements or microbial measurements can shed light on the degree of leaching versus mineralization (Boulton & Boon, 1991; Schreeg et al., 2013).

Even though TBI is subjected to many of the caveats that other litter bag studies are (Boulton & Boon, 1991), it remains an easy, reproducible way to obtain highly standardized measurements by both professional scientists and citizen scientists. Moreover, tea bags could potentially help to disentangle the environmental variables that drive leaching. Future litter decomposition and leaching studies will improve by careful interpretation of solid experiments, by being transparent about definitions used and by explaining the way in which leaching corrections were applied (if any). Comparison across studies is further enhanced by standardization of the methods used, and as outlined above, a correction for leaching is not advised in TBI.

## AUTHOR CONTRIBUTIONS

**Judith M. Sarneel:** Conceptualization (equal); data curation (lead); investigation (equal); visualization (lead); writing – original draft (lead); writing – review and editing (lead). **Janna M. Barel:** Conceptualization (equal); investigation (supporting); writing – review and editing (equal). **Sarah Duddigan:** Conceptualization (equal); writing – review and editing (equal). **Joost A. Keuskamp:** Conceptualization (equal); writing – review and editing (equal). **Ada Pastor:** Conceptualization (equal); writing – review and editing (equal). **Taru Sanden:** Conceptualization (supporting); writing – review and editing (equal). **Gesche Blume‐Werry:** Conceptualization (equal); investigation (lead); writing – review and editing (equal).

## CONFLICT OF INTEREST STATEMENT

We declare no conflict of interest.

## Data Availability

Data obtained from literature sources (Blume‐Werry et al., 2021; Djukic et al., 2018; Friesen et al., 2018; Harmon, 2016; Jiang et al., 2016; Keuskamp et al., 2013; Lind et al., 2022; Mori et al., 2021; Pouyat et al., 2017; Seelen et al., 2019; Thomas et al. (2023 and data file nr. 179 in the TBI database); Xiong & Nilsson, 1997). Data of the incubation was uploaded on Zenodo (10.5281/zenodo.7656594).

## APPENDIX 1

### Extended details on the experiment

We set up a short term incubation in order to test that (1) primary leaching occurs in pulses, following changes in the environment and (2) that after a few days, it already becomes difficult to separate if observed mass losses are due to primary leaching, secondary leaching and/or mineralization. We therefore determined the short‐term (<7 days) mass loss dynamics in soils after rain events that occurred at different time intervals. For this, 40 Lipton green tea bags (biodegradable bags) were individually placed at 4 cm depth in (7.5 × 7.5 × 7 cm) pots filled with a 15:2 mixture of potting soil (k‐jord; NPK 14‐7‐15, Hasselfors Garden AB) and quartz sand (Tärnsjö Grus AB). In order to align with the measurements by Lind et al. (2022), the soil was close field capacity with a soil moisture of 53.2% (flooded soils had a soil moisture of 63.0%). Using 20 pots, we measured green tea mass losses on day 1, 2, 3 and 6 and 7. The remaining 20 pots were used to test the effect of simulated rain events. At day 1, 2 and 6, eight of the pots were ‘rained’ by adding 60 ml of tap water to the pots (which equals a 12 mm rain event, or a wave splash). Four of the rained pots were harvested 1 day after the rain event while the other pots remained untouched until the end of the experiment at day 7 (hence we ‘rained’ only four pots at day 6). The pots were placed in a dark climate room at 18°C. After incubation, the bags were cleaned without water, dried (at least 48 h at 65°C) and weighed to assess mass loss. Differences in weight of the four treatments on day 7 were tested using a one‐way ANOVA. The mass losses due to leaching (the mass loss at day one of the experiment and the differences between the mass observed before and after the rain additions) were tested with a one‐way ANOVA. Tukey post hoc test were done using the ‘emmeans’ package in R version 4.2.2 (R Core Team, 2022). Mass loss in the rained and the control treatment that did not receive rain were compared using a *t*‐test per time‐step using simple linear models.

We observed an initial mass loss of 9% (±0.6 SD) at day one, which is less than the leaching observed in the moist soils by Lind et al. (2022). After addition of water to the pots at day one and two, we observed clear pulses of additional mass loss, which was significantly different in the different rain treatments (*F*_3,12_ = 15.06, *p* < .001). After the first rain event, this was significant more than observed in the same time step in the control without rain, whereas for the other treatments, we did not find significant differences between the mass loss in the rained and not‐rained treatments (Table A1). At day seven, the final mass remaining was highest in the control and lowest in the one rained early, but this difference was not significant (*F*_3,12_ = 1.034 *p* = .412). The soil moisture in the control treatment without rain decreased from 53.2% to 18.5%. In the rain treatments, soil moisture was around 30% (Table A2).

**TABLE A1:** *p*‐Values of the post‐hoc test of the amount of leached material after the rain events.

Leached material	
Contrast	*p*‐Value
Initial‐Early	.020
Initial‐Middle	.927
Initial‐Late	.037
Early‐Middle	.007
Early‐Late	.000
Middle‐Late	.104

Mass loss per time step			
Time	Contrast	*F*_1,6_	*p*
Early	No rain—Early rain	39.33	.001
Middle	No rain—Middle rain	3.975	.093
Late	No rain—Late rain	0.742	.422

**TABLE A2:** Soil moisture in the different treatments measured with a ThetaProbe ML2x (Delta‐T Devices). Measurements were done after removing the tea bags to prevent puncture of the bags.

	Day 0	Day 3	Day 7
No rain	53.2 ± 1.17	27.8 ± 2.48	18.35 ± 3.45
Early rain			23.85 ± 1.67
Middle rain		42.25 ± 1.39	30.775 ± 0.90
Late rain			36.725 ± 0.67

Our simple experiment shows that a sudden increase or pulse in soil moisture (for instance due to rain) can induce considerable additional leaching. The mass lost during the first rain event was higher than the amount lost at the start of the experiment. This shows that rain events potentially can contribute significantly to leaching of primary material, or alternatively, that very short‐term microbial activity can mobilize additional material that is leached upon raining. Both indicate complications with leaching measurements. That is, although shorter durations of leaching experiments increase the likelihood that they only quantify primary leaching, they result in a higher probability of missing events that contribute to primary leaching. For practical reasons, field leaching experiments frequently use a 24 h duration. However, when rain occurring only 24 h after the start of the incubation already leaches some (measurable) secondary material produced during microbial activity, leaching measurements of this duration may already overestimate primary leaching.

For the leaching observed after rain at day 2 or day 3, we can only hypothesize that secondary leaching starts to play a role as we saw some fungi developing under the tea bags. When rain occurs longer after the start of the incubation (such as at day 6 of our experiment) it is more likely that the observed mass loss is a mixture of primary compounds, secondary compounds, and mineralization. In the field, there often is considerable daily variation in temperature and moisture, even in climates with relatively constant weather, which will affect primary leaching. Furthermore, since we observed considerable pulses in rather wet conditions, it is likely that pulsed leaching events are even more common and substantial in drier soils than the ones in our experiment. However, our experiment shows that even under relatively wet conditions, the pulses can be considerable. This means that if we would have followed the recommendation of Lind et al. (2022) and corrected for leaching according to the wetness of our target soil (15% initial mass loss), we would have missed the additional, likely primary, leaching that could occur if a rain event follows shortly after placing them in the field.
